# Comparison of the Regulation of β-Catenin Signaling by Type I, Type II and Type III Interferons in Hepatocellular Carcinoma Cells

**DOI:** 10.1371/journal.pone.0047040

**Published:** 2012-10-04

**Authors:** Wei Li, Xiaojie Huang, Hongfei Tong, Yuxuan Wang, Tong Zhang, Wen Wang, Lili Dai, Tongzeng Li, Shengzhang Lin, Hao Wu

**Affiliations:** 1 Department of Infectious Diseases, Beijing You’an Hospital, Capital Medical University, Beijing, China; 2 Department of General Surgery, Second Affiliated Hospital, Wenzhou Medical College, Wenzhou, Zhejiang, China; 3 Department of Hepato-biliary-pancreatic Surgery, First Affiliated Hospital, Zhejiang University School of Medicine, Hangzhou, Zhejiang, China; 4 Materials Science and Engineering Program, State University of New York at Binghamton, Binghamton, New York, United States of America; University of Hong Kong, Hong Kong

## Abstract

**Background/Objective:**

IFNs are a group of cytokines that possess potent antiviral and antitumor activities, while β-catenin pathway is a proliferative pathway involved in carcinogenesis. Interaction between these two pathways has not been well elaborated in hepatocellular carcinoma (HCC).

**Methods:**

HCC cell lines, HepG2 and Huh7, were used in this study. β-catenin protein levels and corresponding signaling activities were observed by flow cytometry and luciferase assay, respectively. Cell proliferation was quantified by counting viable cells under microscope, and apoptosis by TUNEL assay. DKK1 and GSK3β levels were determined by flow cytometry. Secreted DKK1 was tested by ELISA. FLUD, S3I and aDKK1 were used to inhibit STAT1, STAT3 and DKK1 activities, respectively.

**Results:**

Our findings show that all three types of IFNs, IFNα, IFNγ and IFNλ, are capable of inhibiting β-catenin signaling activity in HepG2 and Huh7 cells, where IFNγ was the strongest (p<0.05). They expressed suppression of cellular proliferation and induced apoptosis. IFNγ expressed greater induction ability when compared to IFNα and IFNλ (p<0.05). All tested IFNs could induce DKK1 activation but not GSK3β in HepG2 and Huh7 cells. IFNs induced STAT1 and STAT3 activation but by using specific inhibitors, we found that only STAT3 is vital for IFN-induced DKK1 activation and apoptosis. In addition, DKK1 inhibitor blocked IFN-induced apoptosis. The pattern of STAT3 activation by different IFNs is found consistent with the levels of apoptosis with the corresponding IFNs (p<0.05).

**Conclusions:**

In hepatocellular carcinoma, all three types of IFNs are found to induce apoptosis by inhibiting β-catenin signaling pathway via a STAT3- and DKK1-dependent pathway. This finding points to a cross-talk between different IFN types and β-catenin signaling pathways which might be carrying a biological effect not only on HCC, but also on processes where the two pathways bridge.

## Introduction

Hepatocellular carcinoma (HCC) is known to be the third most common cause of cancer-related death in the world. Its prevalence is peaking rapidly compared to other cancers possibly due to a high prevalence of hepatitis B and C viral infections [1], [2], [3].

Interferons (IFNs) are a group of pleiotropic cytokines involved in anti-microbial and anti-tumor immunity by enhancing antigen presentation through major histocompatibility complex (MHC) class I and class II interactions, regulating a variety of genes, and facilitating pre-apoptotic responses of infected cells [4], [5].

The significant effects of most IFNs are mediated by the JAK-STAT (Janus Kinase -Signal Transducer and Activator of Transcription) signaling pathway [6], [7]. IFN signaling through JAK-STAT requires an initial step of IFN binding to its receptor, leading to oligomerization of IFN-receptor subunits, which subsequently results in phosphorylation and activation of different combinations of JAK and STAT family proteins. STATs then forms either a homodimer or a heterodimer, and either of which is translocated to the nucleus, where it binds interferon-stimulated response element (ISRE) or γ-activated sequences (GAS) in the promoter region(s) of IFN-regulated genes, which interacts with other transcriptional factors, such as breast cancer susceptibility gene 1 (BCA1) and mini-chromosome maintenance protein 5 (MCM5), and finally regulates IFN-responsive genes. Approximately 500 genes are regulated through IFN-induced JAK-STAT pathway, including IFN-inducible protein 10 (IP-10), GTPase, and suppressor of cytokine signaling I (SOCS-1) [6], [7].

The β-catenin is a key component of the canonical pathway of β-catenin (Wnt) signaling. It is an essential regulator of proliferation, differentiation, and carcinogenesis [8], [9]. The canonical β-catenin pathway is briefly initiated by the binding of Wnt proteins to their receptor (Frizzled, Fz) and low-density lipoprotein receptor-related protein 5 or 6 (LRP 5/6) co-receptors. This consequently inhibits the β-catenin destruction complex (GSK3β, axin, APC, and Ck1), leading to accumulation of activated β-catenin. Along with co-activators (p300 and CBP), active β-catenin activates T-cell factor/lymphoid enhancer (TCF/LEF) transcription factors and finally aims at the target gene transcriber [8]. Active β-catenin could also bind to cadherins in the cell membrane along with actin to provide structural support for adhesion [10], [11].

Interruption of apoptosis is a crucial step in the development of cancer [12]. Therapeutic activation of apoptosis in cancer cells is a potential anticancer strategy [12]. More than three hundred IFN-regulated genes are involved in apoptosis [3], [13], [14], [15]. Previous studies have demonstrated the antitumor potential of IFNs in various cancer cells [13]. Even the latest type III IFNs, IFNλs, have been proven to be able to activate downstream signaling in selected cancer cells [16]. But the detailed mechanisms on how different IFNs induce apoptosis and how the interplay between IFN signaling and β-catenin pathway is accomplished in HCC cells have not yet been reported.

In this study we aimed to find out and compare the effects and mechanisms of IFNα, IFNγ and IFNλ in suppressing β-catenin signaling and promoting apoptosis induction in HCC cells. Our study demonstrates different characteristic potentials of various types of IFNs, and unfolds a link between IFNs and β-catenin signaling pathways that impact apoptosis induction in HCC cells and may have a larger biological impact on mechanisms of carcinogenesis in liver, and benefit liver cancer therapy.

## Materials and Methods

### Cell Lines and Reagents

The hepatocellular carcinoma cell lines HepG2 and Huh7 were obtained from PriCell Research Institute. They were propagated in DMEM (Gibco Invitrogen, Carlsbad, CA) supplemented with 10% heat-inactivated FBS (Sigma, St. Louis, MO) and 1% penicillin-streptomycin (Gibco Invitrogen). The cells were used at approximately 80% confluence. Human recombinant IFNγ, GSK3β antibody (pY216), pSTAT1 (S727)-AF647 mAb, pSTAT3 (pY705)-AF488, Caspase3-AF488 were purchased from BD Pharmingen (San Jose, CA). APC and FITC-conjugated goat anti-mouse antibodies and FITC-bovine anti-goat IgG (H+L) antibody were purchased from Jackson ImmunoResearch Lab Inc (West Grove, PA). Human recombinant IFNα, IFNλ (IFNλ1, IL29) and Fludarabine (FLUD) were purchased from Sigma-Aldrich (St. Louis, MO). STAT3 inhibitor V, Stattic, STAT5 inhibitor, and GSK3β inhibitor IX were purchased from CalBiochem/EMD biosciences (Gibbstown, NJ). hDKK1 neutralizing antibody was purchased from R&D Systems (Minneapolis, MN) while DKK1 detection antibody was purchased from Abcam (Cambridge, MA). DKK1 ELISA was purchased from RayBiotech (Norcros, GA) and used as recommended.

### DNA Constructs and Transfection

HepG2 and Huh7 hepatocellular carcinoma cell lines were transiently transfected using the LT-1 transfection reagent (Mirus Bio LLC; Madison, WI) as recommended by the manufacturer. To measure β-catenin-dependent signaling activity, 5×10^6^ cells were transfected with 10 µg TOPflash reporter construct (Millipore; Billerica, MA). TOPflash construct consists of two sets of three TCF/LEF binding sites linked to a luciferase reporter. The cells were also co-transfected with 1 ng *Renilla* construct (Promega, Madison, WI) to normalize for transfection efficiency and green fluorescent protein (GFP) (pMaxGFP, Lonza, Biologics, Portsmouth, NH) to equalize the amount of total DNA used per transfection condition. Firefly and Renilla luciferase activity were measured using dual luciferase assay reporter system (Promega, Madison, WI).

### Immunofluorescence Staining and Flow Cytometry Analysis

To detach cells without cleaving surface proteins, cells were incubated with 1 mM EDTA for 5 minutes then washed and suspended in 1×PBS. Cells were stained with appropriate target antibodies and isotype antibodies using conventional surface and/or intracellular staining methods. When both surface and intracellular staining were prepared, cells were first fixed and made permeable using BD Cytofix/Cytoperm,Fixation and Permeability Solution (BD Pharmingen, San Jose, CA), followed by staining for intracellular proteins. Cells were then washed extensively with 1×PBS in order to remove excess antibodies, stained for extracellular targets, and fixed with 2% formaldehyde. Fluorescence was evaluated with a FACSCalibur flow cytometry and data analyzed using FlowJo software (TreeStar, Ashland, OR).

### Proliferation and Cell Viability Assays

Cell viability assays were performed as described previously [17]. Briefly, to determine cell viability, equal amount of cells (10^5^/well) were plated in wells of 6-well plates and transfected and/or treated, as indicated in the text. Dead cells lost their attachment and were washed away by 1×PBS. Viable (adherent) cells were released from the wells by trypsinization before cell counting.

### TUNEL Assay

TUNEL assay to determine DNA fragmentation in apoptotic cells was performed according to the manufacturer’s suggested protocols (Promega). 3–5×10^6^ cells were briefly trypsinized, washed twice with cold PBS, fixed in 4% paraformaldehyde at 4°C for 20 minutes, and then washed again with PBS and made permeable with 0.5 ml 0.5% saponin at 22°C for 5 minutes. The cells were washed with PBS, incubated with 80 µl equilibration buffer at 22°C for 5 minutes, washed with PBS, re-suspended in 50 µl Nucleotide Mix and incubated in the dark at 37°C for 1 hr. Cells were washed again with PBS and analyzed by fluorescence microscopy.

### Statistical Analysis

Statistical analyses were performed using Prism software (GraphPad Prism, San Diego, CA). Untreated and treated groups were compared using the Student's *t*-Test with the data normally distributed. Abnormally distributed data had the two groups compared using the non-parametric Mann-Whitney test. All tests were two-tailed and a p-value <0.05 was considered significant.

## Results

### IFNα, IFNγ and IFNλ Suppress β-catenin Level in HepG2 and Huh7 Cells

**Figure 1:**
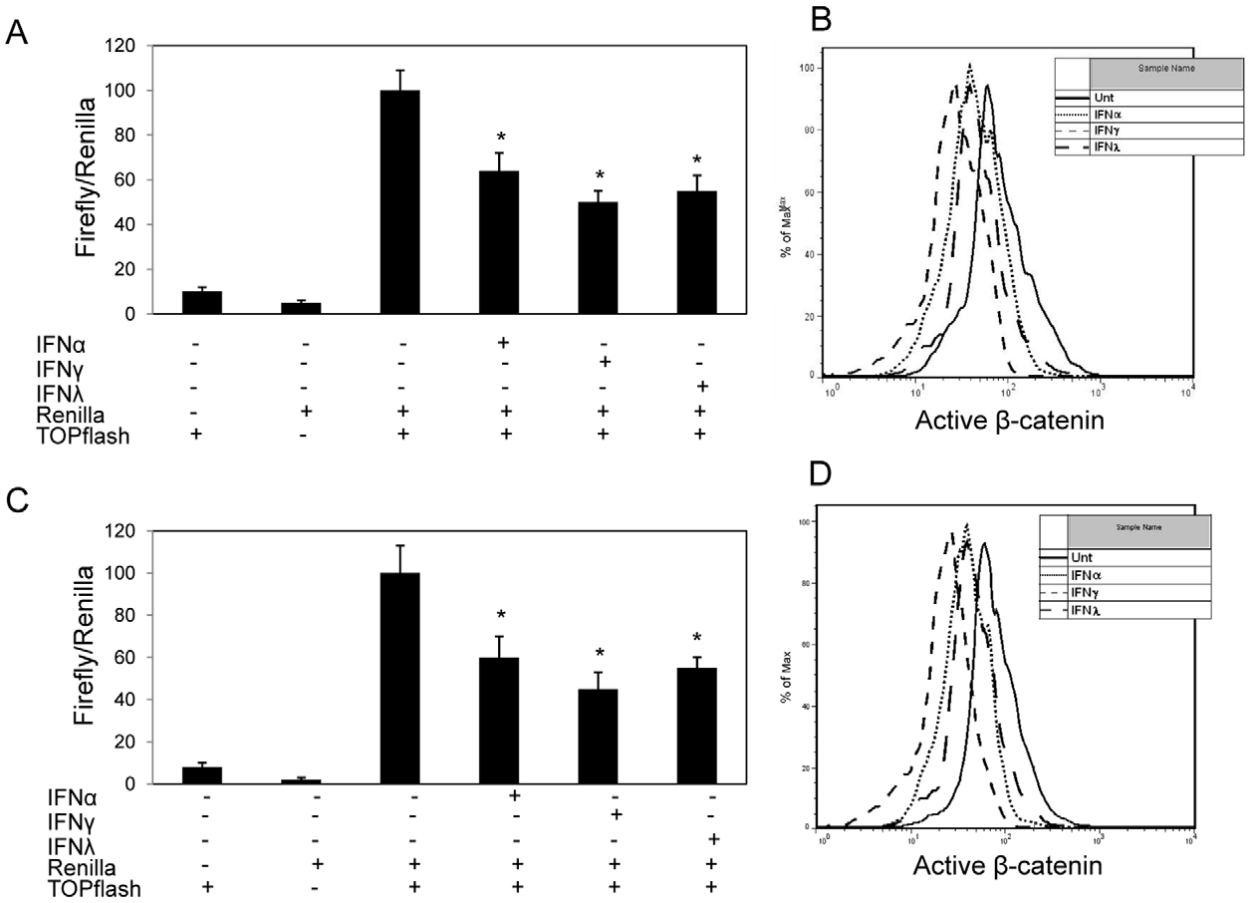
IFNs down-regulate β-catenin signaling pathway. HCC cell lines, HepG2 (**A**) and Huh7 (**C**), were left untreated or treated with IFNα (100 ng/ml), IFNγ (100 ng/ml) or IFNλ (100 ng/ml) for 24 hrs prior to transfection with TOPflash luciferase and Renilla luciferase constructs. After resting for 4 hrs, the cells were cultured with or without initial treatment of different IFNs. Dual luciferase activity was measured 24 hrs later. Data shown is normalized to Renilla activity. HepG2 (**B**) and Huh7 (**D**) were treated with or without IFNα (100 ng/ml), IFNγ (100 ng/ml) or IFNλ (100 ng/ml) for 48 hrs and expression of hypophosphorylated/active β-catenin level was measured by conventional intracellular flow cytometry. Data represent a minimum of three experiments and asterisks denote p<0.05 in comparison to untreated samples.

We evaluated whether IFNα, IFNγ and IFNλ down-regulate β-catenin in hepatocellular carcinoma (HCC) cell lines, HepG2 and Huh7. HepG2 and Huh7 cells were co-transfected with a TCF/LEF firefly luciferase construct (TOPflash) and a control reporter (Renilla luciferase), and then left untreated or treated with IFNα (100 ng/ml), IFNγ (100 ng/ml) and IFNλ (100 ng/ml), respectively. The TOPflash reporter is an indicator of basal and inducible levels of β-catenin-dependent signaling. At 24 hrs post IFN treatment, IFNα, IFNγ and IFNλ markedly reduced β-catenin signaling by approximately 36%, 55% and 50%, respectively. And the reduction in Huh7 cells is comparable to that observed in HepG2 (**Fig. 1A, C**). IFN-mediated inhibition of β-catenin signaling in HepG2 and Huh7 cells was also consistent with reduction in active hypophosphorylated β-catenin, as evaluated by intracellular flow cytometry (**Fig. 1B, D**). It is clear that all three types of IFNs are capable of inhibiting β-catenin signaling in HCC cell lines, and IFNγ is the most potent among the three studied IFNs (p<0.05).

### IFNα, IFNγ and IFNλ Inhibit Proliferation and Induce Apoptosis in HCC Cell Lines

**Figure 2:**
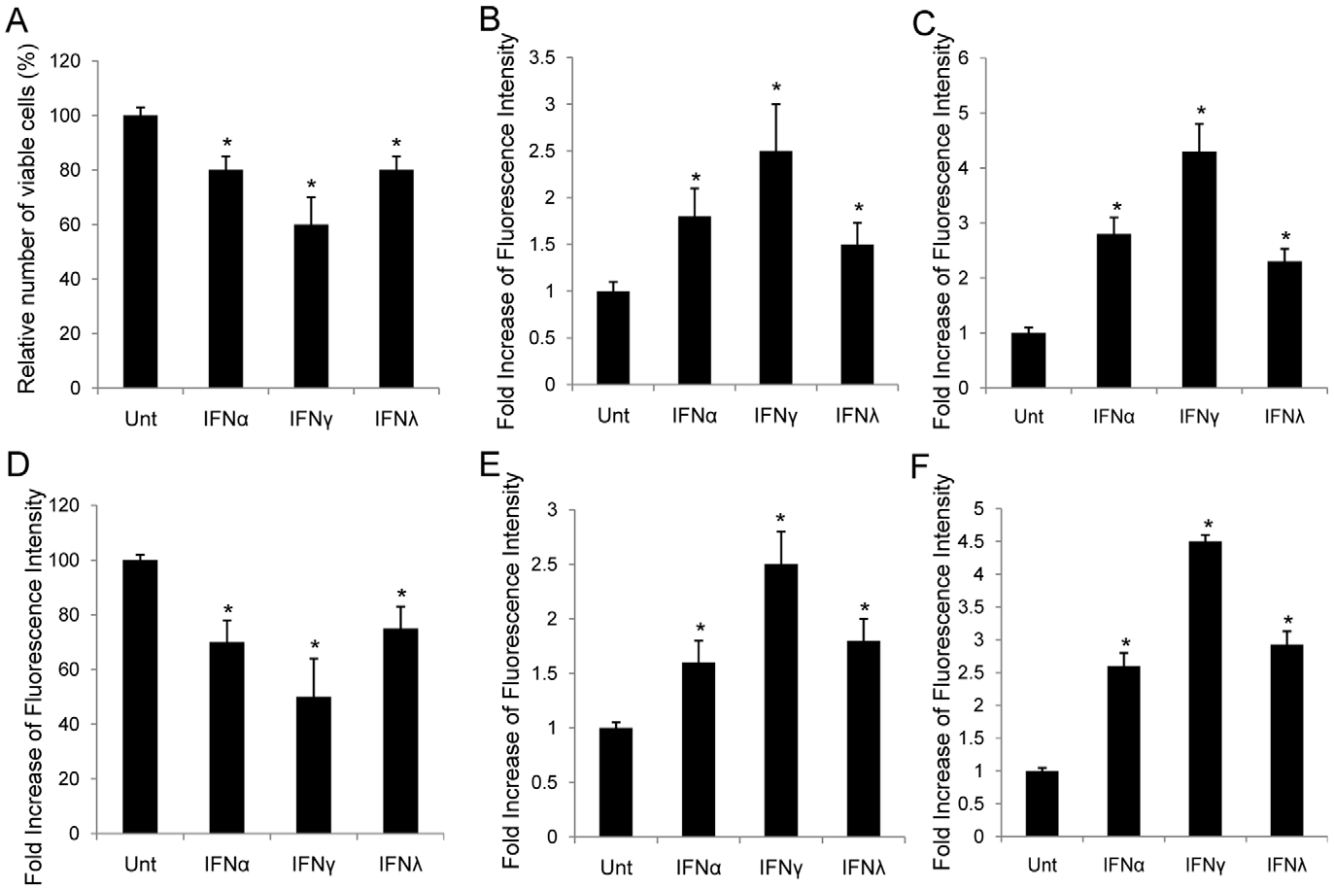
Impact of IFN-mediated β-catenin regulation on proliferation and apoptosis in HCC. HepG2 (**A**) and Huh7 (**D**) cells were left untreated or treated with IFNα (100 ng/ml), IFNγ (100 ng/ml) or IFNλ (100 ng/ml) for 72 hrs. Cell viability was determined at 72 hrs. The abscissa represents the types of stimulation. The ordinate represents percentage of live cells relative to the mock treated cells. Apoptosis in HepG2 (**B, C**) and Huh7 (**E, F**) was measured by TUNEL assay and flow cytometry targeting active caspase 3. The ordinate represents fold increase of fluorescence intensity relative to the untreated cells. Data represent a minimum of three experiments and asterisks denote p<0.05 in comparison to untreated samples.

To evaluate the effects of IFNs on the proliferation of hepatocellular carcinoma cell lines, HepG2 and Huh7 cells were left untreated or treated with IFNα (100 ng/ml), IFNγ (100 ng/ml) and IFNλ (100 ng/ml) for 72 hrs, and viable cells were counted under microscope. The results demonstrated that, if compared with untreated controls, IFNα, IFNγ and IFNλ reduced viable cell counts in HepG2 cells by approximately 20%, 40%, and 20%, respectively, where IFNγ was the strongest (p<0.05) **(** **Fig. 2A** **)**. Similar pattern was observed in Huh7 cells **(** **Fig. 2D** **)**.

To determine whether apoptosis was induced by IFNs in HepG2 cells, TUNEL assay was used to detect DNA fragmentation in apoptosis, and was performed on HepG2 cells, treated with or without different IFNs for 72 hrs, respectively. The fluorescence was increased by 1.8-, 2.5-, and 1.5-folds when treated with IFNα, IFNγ and IFNλ, respectively. IFNγ was still the most potent among the three IFNs (p<0.05) **(** **Fig. 2B** **)**. Apoptosis was confirmed via detecting activation of caspase 3 by flow cytometry using specific antibody against activated caspae 3 **(** **Fig. 2C** **)**. Anti-proliferative effects and apoptosis induction by different IFNs in HepG2 cells were similar to those in Huh7 cells, except that the apoptosis induced by IFNα was stronger than that of IFNλ in HepG2 cells, while it expressed contrary to that found in Huh7 cells (p<0.05) **(** **Fig. 2D–F** **)**. IFNγ was always the most potent one when compared with IFNα and IFNλ in both cell lines (p<0.05). Apparently, IFNs suppressed cell proliferation and induced apoptosis in HCC cell lines.

### IFNα, IFNγ and IFNλ Induced DKK1, but not GSK3β

**Figure 3:**
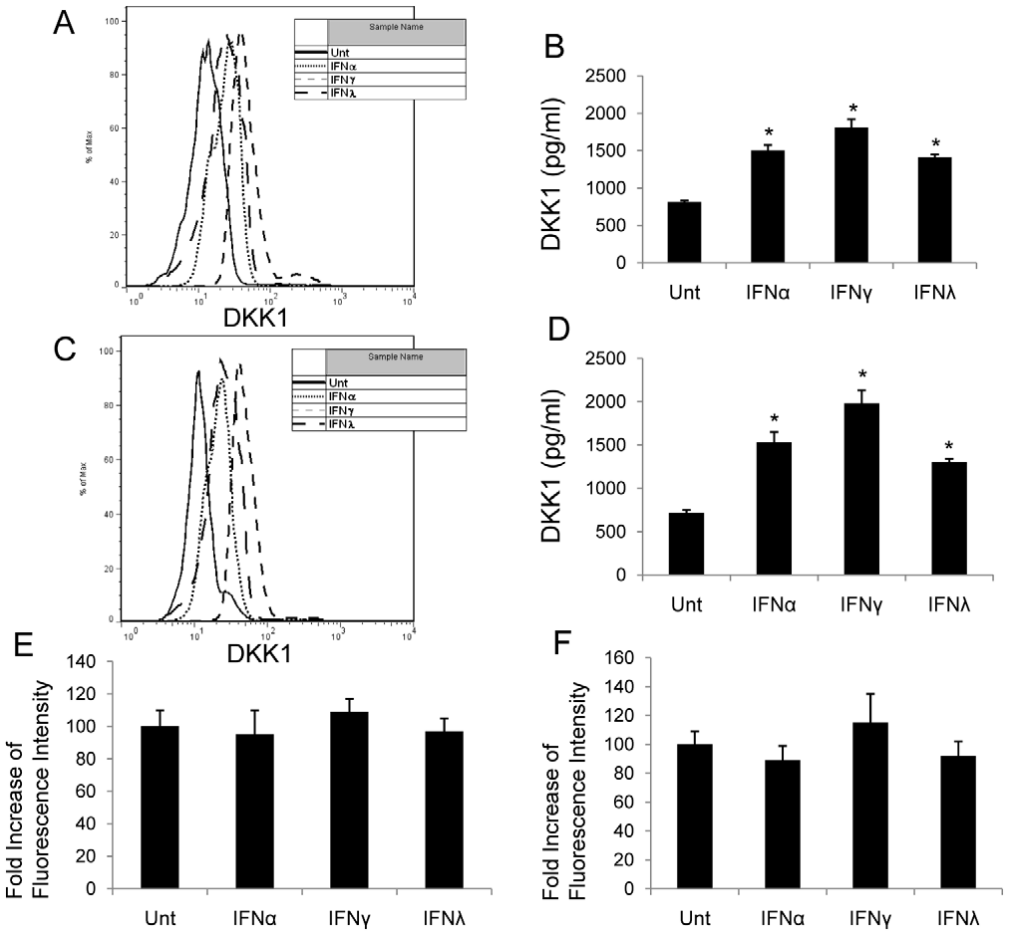
IFNs induce DKK1 expression and have no effects on GSK3β. HepG2 and Huh7 cells were treated with or without IFNα (100 ng/ml), IFNγ (100 ng/ml) or IFNλ (100 ng/ml) and expression of DKK1 was measured by flow cytometry (**A**, **C**) or ELISA (**B**, **D**) at 48 hrs post-treatment. Active GSK3β level was measured in HepG2 and Huh7 cells at 48 hrs post different IFN exposure by flow cytometry (**E**, **F**). Data represent a minimum of three experiments and asterisks denote p<0.05 in comparison to untreated samples.

To determine how IFNs down-regulate β-catenin signaling activity, we evaluated the impact of different IFNs on two potent endogenous antagonists of the β-catenin pathway, DKK1 and GSK3β. DKK1 antagonized β-catenin signaling by depleting frizzled co-receptors (LRP) and thus, inhibiting frizzled activation through Wnt ligands. Following CK1-mediated phosphorylation of β-catenin at serine 45, GSK3β phosphorylates β-catenin at Thr 41, Ser 33, and Ser 37, which tags β-catenin for ubiquitination by βTrCP and proteomic degradation [18]. IFN-treated HCC cells demonstrated a significant induction in DKK1 expression as observed by both flow cytometry and ELISA and was consistent between HepG2 and Huh7 cells. The levels of DKK1 induction was the highest by IFNγ treatment when compared with that of IFNα and IFNλ (p<0.05, respectively) (**Fig. 3A–D**). Active GSK3β expression was not induced by any IFN stimulation in either HepG2 or Huh7 cells (**Fig. 3E, F**).

### IFN Induced-apoptosis in HCC is Dependent on their Ability to Induce DKK1 and STAT3

**Figure 4:**
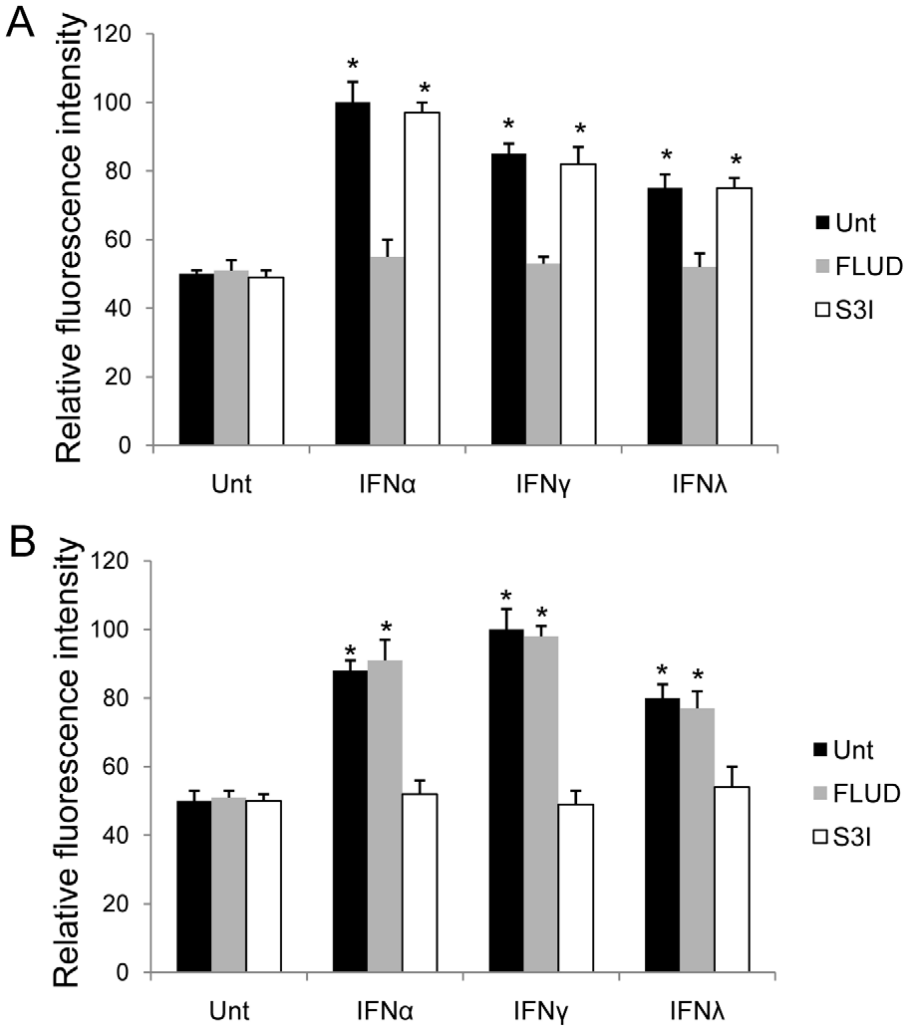
IFNs induce activation of STAT1 and STAT3 in HCC. HepG2 cells were left untreated or treated with IFNα, IFNγ or IFNλ for 0.5 hr in the absence (black bars) or presence of STAT1 inhibitor (FLUD, grey bars) or STAT3 inhibitor (S3I, open bars), and active STAT1 (**A**) and STAT3 (**B**) levels were measured by flow cytometry. Data represent a minimum of three independent experiments. Asterisks denote p<0.05 in comparison to untreated samples.

JAK-STAT pathway is used by all IFNs to conduct downstream signaling, where STAT family proteins are induced selectively by different IFNs. Among proteins in the STAT family, STAT1 and STAT3 can be activated by all types of IFNs [7]. We first tested the activation of STAT1 and STAT3 by IFNα, IFNγ and IFNλ in HepG2 by flow cytometry, and results demonstrated that all three IFNs induced STAT1 and STAT3, among which IFNα was strongest in STAT1 activation, and IFNγ was most potent in STAT3, while IFNλ seemed to be the weakest in both (p<0.05, respectively). The pattern of STAT3 activation, but not STAT1, by different IFNs was consistent to the intensity of apoptosis induction with corresponding IFNs (**Fig. 4** **, black bars**). Similar results were observed in Huh7 cells (data not shown).

**Figure 5:**
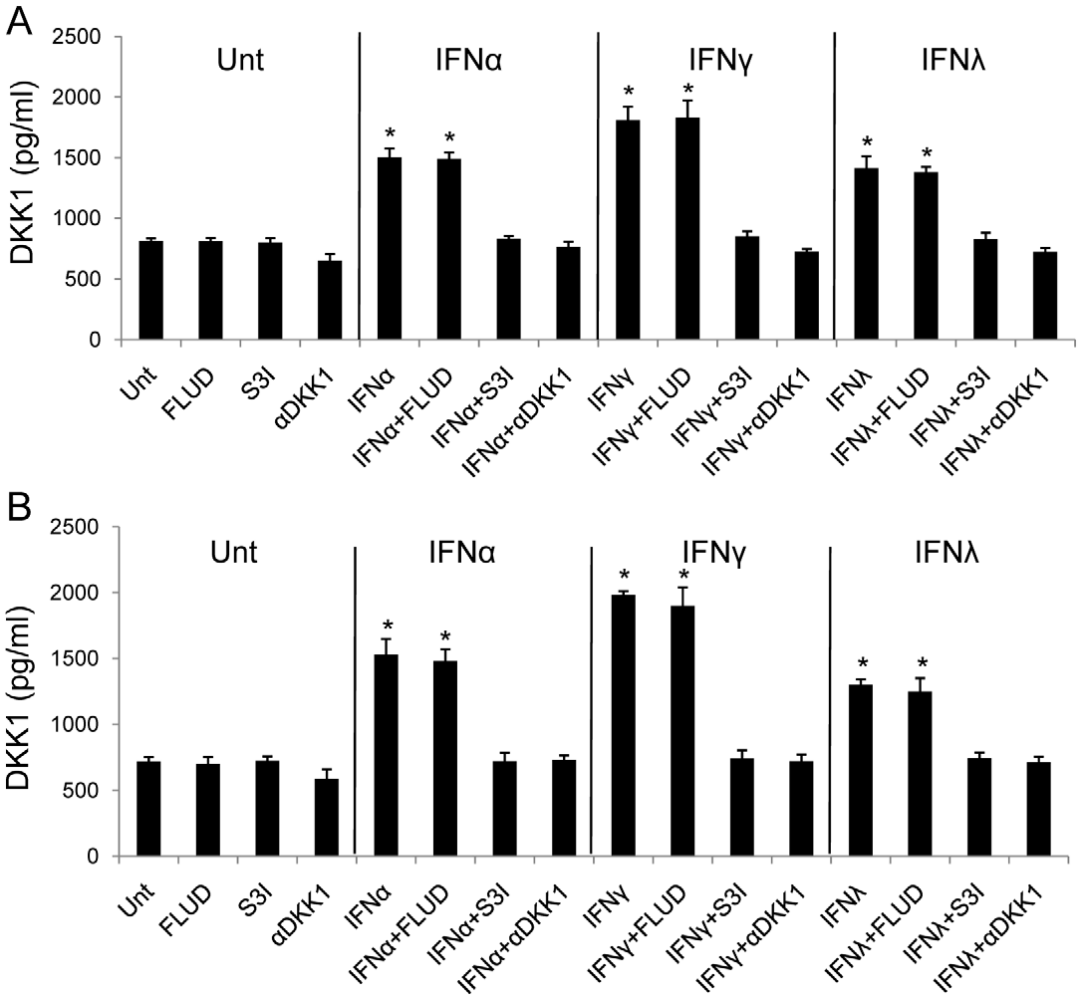
IFN-induced up-regulation of DKK1 is dependent on STAT3 activation. HepG2 (**A**) and Huh7 (**B**) cells were left untreated or treated with FLUD, S3I, or αDKK1 (DKK1 neutralizing antibody) alone or in combination with IFNα, IFNγ or IFNλ, respectively for 48 hrs. DKK1 levels were measured by ELISA. Data represent a minimum of three independent experiments. Asterisks denote p<0.05 in comparison to untreated samples.

Testing for the necessity of DKK1, STAT1 and STAT3, we chose to use a neutralizing antibody targeting DKK1 (αDKK1), STAT1 inhibitor (FLUD), and STAT3 inhibitor (S3I). Addition of neutralizing antibody against DKK1 abrogated the ability of IFNs to induce DKK1 and reduced the level of DKK1 from untreated cultures in both HepG2 and Huh7 cells (**Fig. 5**). These data suggest that HCC cell lines constitutively express DKK1, which are consistent with the knowledge that DKK1 is a target gene of the β-catenin pathway and regulates the expression of this pathway in a feedback loop mechanism [8]. We next tested for the effects of FLUD and S3I in IFNα, IFNγ and IFNλ signaling pathways. FLUD and S3I effectively inhibited STAT1 and STAT3 activation, respectively, in HepG2 (**Fig. 4** **, grey and open bars**) and Huh7 cells (data not shown). IFN-induced DKK1 in HepG2 and Huh7 cells were abrogated by S3I, but not FLUD, indicating that STAT3 but not STAT1, is required for IFN-induced DKK1 up-regulation (**Fig. 5**). It is clear that STAT3 is upstream to DKK1 in IFN signaling cascade in HCC cells and is indispensable to IFN-induced DKK1 induction.

**Figure 6:**
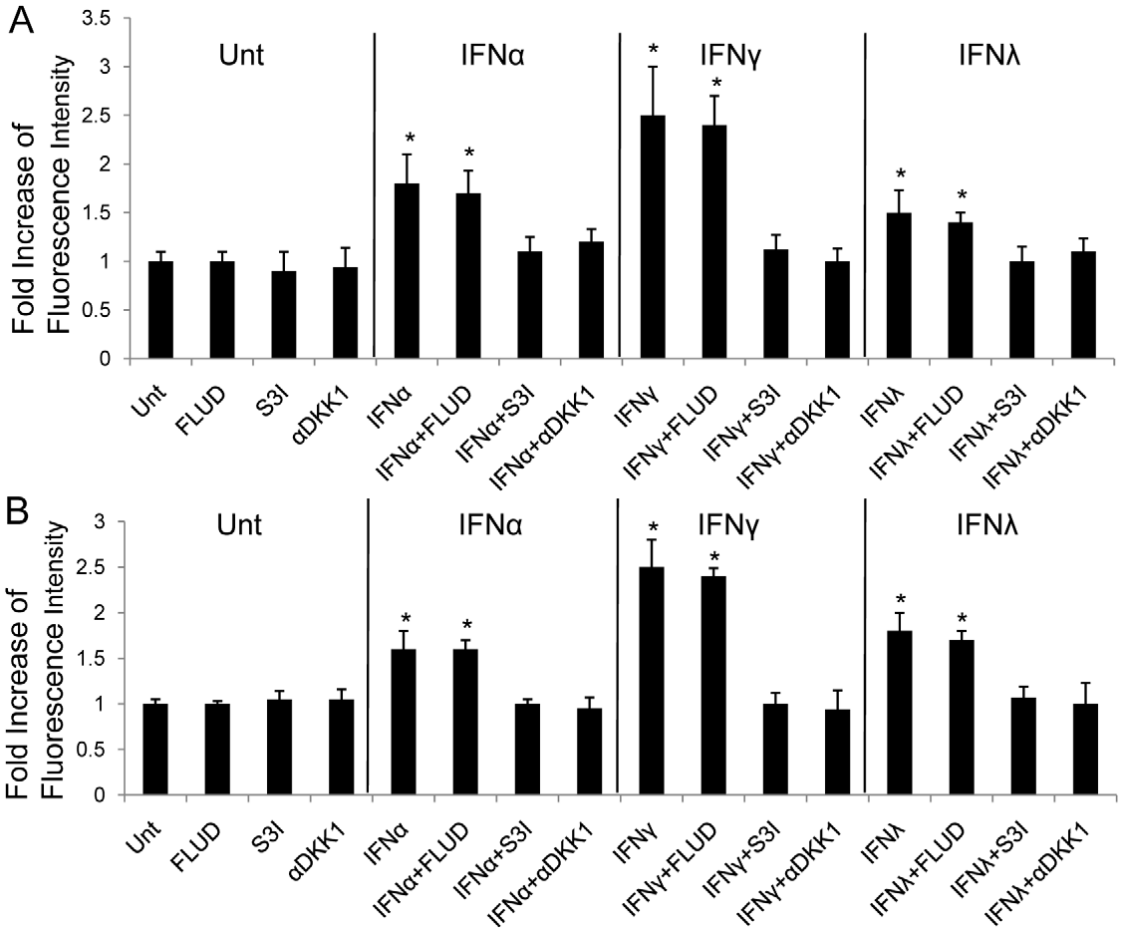
IFN-induced apoptosis in HCC is DKK1 and STAT3-dependent. HepG2 (**A**) and Huh7 (**B**) cells were left untreated or treated with FLUD, S3I, or αDKK1 alone or in combination with IFNα, IFNγ or IFNλ, respectively for 72 hrs. The levels of induced apoptosis were measured by TUNEL assay. Data represent a minimum of three independent experiments. Asterisks denote p<0.05 in comparison to untreated samples.

Since DKK1, STAT1 and STAT3 could be induced by all three types of IFNs, we next tested the contribution of DKK1, STAT1 and STAT3 to IFN-induced apoptosis in HCC cell lines. Addition of αDKK1 or S3I abrogated the capability of IFNα, IFNγ and IFNλ to induce apoptosis in HepG2 and Huh7 cells, determined by TUNEL assay. However, FLUD showed no effects on blocking IFN-induced apoptosis, indicating that STAT1, although activated by IFNs, had no role in this regulation (**Fig. 6**). These data suggest that all IFN-induced apoptosis in HCC cell lines is DKK1 and STAT3-dependent. Collectively, these findings demonstrate an interaction between two prominent signaling pathways, β-catenin and IFN signaling, that interface with each other to impact the outcome of hepatocellular carcinoma.

## Discussion

IFN signaling, as a critical component of the innate immunity, has a broad effect on the immune system. Canonical β-catenin signaling is involved in cell proliferation and differentiation. These two pathways have been reported to have a cross-regulation at certain levels. Nava et al discovered that IFNγ exerted a biphasic effect on intestinal epithelial cell proliferation and apoptosis by regulating DKK1, and in turn β-catenin signaling [19]. Last year, we reported the interface of IFNγ signaling and β-catenin signaling via up-regulation of STAT3 and DKK1 in brain astrocytes and its impact on HIV replication [20]. On the other hand, level changes of β-catenin also can affect IFN signaling [21]. There is also a report indicating that *in vitro* and *in vivo* pegylated-IFNα treatment leads to increased export of nuclear β-catenin in multiple HCC cell lines [22]. And in preneoplastic rat livers, *in vivo* IFNα2b treatment inhibits Wnt/β-catenin/TCF pathway and promotes programmed cell death possibly providing a link with FOXO (forkhead box containing protein class O) pathway [23]. Additional study shows that IFNα2b attenuated Wnt/β-catenin signal by decreasing β-catenin and Frizzled7 receptor proteins contents and the interaction of β-catenin with TCF4, in HCC cell lines [24]. It is clear that there is a cross-linked interaction between IFN and β-catenin signaling, but as to how and under which circumstances still remain unclear. Here in this present study, we investigated the regulation of all three types of IFNs, including the latest type III interferons (IFNλs), on β-catenin signaling and downstream induced apoptosis in HCC.

Our results demonstrated that, in different HCC cell lines, all three types of IFNs suppress the level of activated β-catenin and β-catenin signaling activity. The result of IFNγ is consistent with our findings in astrocytes [20]. Among selected IFNs, IFNγ was the strongest suppressor if compared with the other two IFNs (p<0.05) (**Fig. 1**). IFNα, IFNγ and IFNλ could inhibit cell proliferation and induce apoptosis in HCC cells, and this is confirmed by our data (**Fig. 2**). By comparison, we found that IFNγ was the most potent among all three types of IFNs in terms of apoptosis induction (**Fig. 2**). Since all IFNs share similar downstream signaling pathway, the JAK-STAT pathway [7], it is reasonable that they all inhibit β-catenin and promote apoptosis. We tested STAT1 and STAT3 activation by different IFNs and found that both STATs could be activated. But by using specific STAT inhibitors, we demonstrated that only STAT3 was required for IFN-induced apoptosis. And the pattern of STAT3, but not STAT1, showed that, activation by different IFNs was consistent to the intensity of apoptosis induction by corresponding IFNs (**Fig. 4**, **6**). Our data add to the body of evidence pointing to the interface between different IFNs and β-catenin signaling in different cell types, and contribution of STAT1-independent mechanisms to IFN-dependent downstream activities [25], [26]. To our knowledge, there have not been any previous reports about STAT1-independent activities induced by IFNλ. In addition, we showed the impact of this interface on apoptosis induction, which is one of the major approaches to treatment of cancers.

β-catenin pathway is tightly regulated to avoid aberrant activation. A number of proteins in this signaling are regulated at gene level by β-catenin/TCF transcriptional regulation, including DKK1, an important secreted endogenous inhibitor against β-catenin pathway [8]. We found that in tested HCC cell lines, DKK1 could be induced by all three types of IFNs, where IFNγ was the most capable one. IFN-induced DKK1 elevation could be blocked by STAT3 inhibitor, but not by STAT1 inhibitor (**Fig. 5**). Extensive cross-linked interaction exists between the β-catenin pathway and other signal-transduction cascades, including the PI3K/Akt and p38/MAPK pathways, which converge on GSK3β [8]. In this study, no IFN had any effect on GSK3β in HCC (**Fig. 3**). This is consistent with our previous findings in astrocytes [20]. It seems clear that GSK3β is not a target of IFN regulation.

The two cell lines used in this study, HepG2 and Huh7, are not identical in terms of β-catenin signaling. There is a truncated form of β-catenin, which is constitutively active, in HepG2 cells in addition to the wild-type [27], while in Huh7 cells, the mRNA and protein levels of Frizzled7 are much higher than in other cells, such as HepG2 [28]. Despite the difference, in our results, the effects of different IFNs were similar between these two cell lines, except that the apoptosis induced by IFNα was stronger than by IFNλ in HepG2 cells, while it was contrary in Huh7 cells (p<0.05) (**Fig. 2**).

HCC is a devastating disease and among the most fatal cancers in the world. Treatment options are limited mainly due to inefficiency of existing anticancer chemotherapeutic drugs against HCC. IFNα has been used to treat HBV and HCV infections, and also indicated for HCC. In addition, there are many completed and ongoing clinical trials on IFNα treating HCC [5]. Yet its antitumor mechanisms are still not completely clear. IFNγ is generally a regulatory IFN that affects many key mediators of the immune system [5]. IFNλs were discovered only a decade ago and shows great potentials in antiviral and antitumor therapy due to its IFNα-like activities with much fewer side effects because of limited receptor distribution [29], [30]. So far, there are very limited studies to compare the activities induced by these three types of IFNs in HCC, especially for IFNλs, the latest group of IFNs [31]. β-catenin pathway is a surviving signaling and adapted by the oncogenesis of various cancers, including HCC [8], [9]. It is also related to the metastasis of HCC [32]. Since IFNs have demonstrated antitumor activities in HCC, while β-catenin signaling is prosurvival, there is a great possibility that IFN may regulate β-catenin signaling directly or indirectly. But the correlation between the IFN signaling and β-catenin signaling in HCC has not yet been previously reported. We first revealed this interaction. Greater understanding on how IFNs regulate β-catenin signaling may benefit the therapeutic application of different IFNs on HCC management.

Given the link we demonstrate here between IFNs and β-catenin signaling pathway, IFNs may have broader roles than that was previously appreciated. Understanding the interactions between IFNs and β-catenin signaling will have a broader impact not only on carcinogenesis but also on understanding the normal biology of liver cells at the interface of IFNs and β-catenin-dependent pathways.
